# Compact Quantum Magnetometer System on an Agile Underwater Glider

**DOI:** 10.3390/s21041092

**Published:** 2021-02-05

**Authors:** Brian R. Page, Reeve Lambert, Nina Mahmoudian, David H. Newby, Elizabeth L. Foley, Thomas W. Kornack

**Affiliations:** 1 School of Mechanical Engineering, Purdue University, West Lafayette, IN 47907, USA; page82@purdue.edu (B.R.P.); lamber53@purdue.edu (R.L.); 2 Twinleaf LLC, Plainsboro, NJ 08536, USA; newby@twinleaf.com (D.H.N.); foley@twinleaf.com (E.L.F.); kornack@twinleaf.com (T.W.K.)

**Keywords:** marine sensors technologies, underwater search and exploration, automated detection, classification, and segmentation of marine objects, marine robotics, marine magnetometry, underwater glider

## Abstract

This paper presents results from the integration of a compact quantum magnetometer system and an agile underwater glider for magnetic survey. A highly maneuverable underwater glider, ROUGHIE, was customized to carry an increased payload and reduce the vehicle’s magnetic signature. A sensor suite composed of a vector and scalar magnetometer was mounted in an external boom at the rear of the vehicle. The combined system was deployed in a constrained pool environment to detect seeded magnetic targets and create a magnetic map of the test area. Presented is a systematic magnetic disturbance reduction process, test procedure for anomaly mapping, and results from constrained operation featuring underwater motion capture system for ground truth localization. Validation in the noisy and constrained pool environment creates a trajectory towards affordable littoral magnetic anomaly mapping infrastructure. Such a marine sensor technology will be capable of extended operation in challenging areas while providing high-resolution, timely magnetic data to operators for automated detection and classification of marine objects.

## 1. Introduction

Underwater magnetic surveys have a long and rich history [1]. Underwater magnetic surveys are typically conducted by moving a magnetometer or gradiometer through water along a series of parallel straight-line tracks within an area of interest. The overall length and separation of the tracks depend on the size of the survey area, and the depth or size of potential targets of interest. Past efforts at marine magnetic survey have been hindered by the size and power consumption of available magnetometers.

Magnetometers have been towed behind surface ships, incorporated in large Remote Operated vehicles (ROVs) and Autonomous Underwater Vehicles (AUVs), and are used for purposes ranging from geophysical and biological research to anthropogenic undersea target detection [2,3,4,5,6,7,8]. For example, anthropogenic targets are strongly suspected if an object is detected in sonar/visual-based methods and has a strong magnetic signal. Shipwrecks, aircraft wrecks, Mine-Like Objects (MLOs) and unexploded ordnance (UXO) can be localized and classified via a fusion of magnetometry, sonar, and visual inspection methods [4,5,9,10,11].

In this work a uniquely low-cost, configurable, autonomous, compact, and agile underwater glider equipped with a cutting edge quantum magnetometer system is presented for search, detection, and classification of marine objects in extreme environments. The proposed system was validated for use in underwater magnetic anomaly detection through the detection of seeded magnetic targets in a confined testing environment. This work is a collaborative effort between Twinleaf LLC and Purdue University. Twinleaf has developed a compact, low-cost, optically pumped scalar atomic magnetometer system. Purdue University has developed a custom-made agile underwater glider called ROUGHIE.

The ROUGHIE’s customizability is leveraged to drastically reduce its magnetic signature while simultaneously integrating a sensor boom. The decrease in the platform’s magnetic signature allows the sensor boom to be integrated close to the vehicle with minimal structural change, thus preserving the platform’s maneuverability. The sensor boom houses a sensor suite comprising of an optical scalar magnetometer and a magneto-restive vector magnetometer. This paper describes the integration of the magnetometer system with the ROUGHIE and experimental validation and data collection for magnetic anomaly detection in an indoor swimming pool with a high noise floor.

The contribution of this work is multi-faceted: specifically, (1) validation of the ability of a small-scale buoyancy-driven vehicle to carry a relatively large sensor suite during nominal data collection flights, (2) the validation of a Glider sensor suite’s ability to localize magnetic anomalies with a small scale underwater glider in constrained environments, and (3) a strategy for identifying and mitigating AUV magnetic signatures to lower the sensing noise floor. This all leads to the eventual end goal of enabling constant, long-term monitoring with timely feedback to human operators through automated detection and classification of marine objects. Further, the integration of such a low-cost, high-performance sensor can form the basis for augmenting prior work on deep learning for recognition of marine objects [12].

The remainder of this paper provides a brief background on fundamentals in Section 2, a description of the experimental setup of the magnetic sensing platform in Section 3, experimental setup in Section 4, the experimental results of platform deployment and testing in Section 5, and finally a brief conclusion of the work is presented in Section 6.

## 2. Background

This paper builds upon existing work on underwater gliders, underwater motion capture, and marine magnetometry.

Underwater gliders are a class of Autonomous Underwater Vehicles (AUVs) that provide both compact size and exceptional endurance, and recent developments have made cost-effective prototypes available for development [13]. Gliders are unique in that they move through the water via changes in buoyancy, and thus can do so with great efficiency and minimal disturbance to the environment. The endurance of underwater gliders is measured in weeks compared to hours or days for conventional AUVs. Underwater gliders are particularly suited for magnetometry, because they spend significant operational time with no motors operating. Due to their relatively quiet and gentle operation, gliders have been used for sensitive acoustic measurements in biology and climate studies [14,15]. Commercial gliders, having not been designed with a magnetic signature in mind, may still generate a significant magnetic signal. Prior magnetometer integration on commercial gliders have required calibration and compensation for the motion of ferromagnetic components within the glider [16,17].

**Figure 1:**
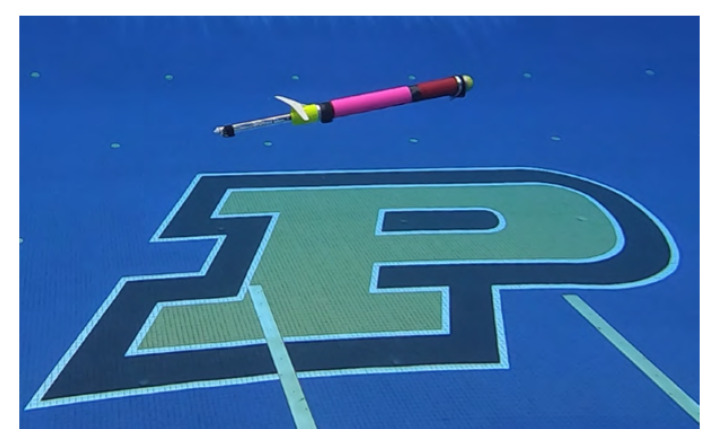
ROUGHIE surfacing during data collection. The magnetometer boom is visible at aft towards the left, with trim weights visible at the end.

Over the past several years the Research-Oriented Underwater Glider for Hands-on Investigative Engineering (ROUGHIE), Figure 1, has been developed as a modular, low-cost, and highly-maneuverable underwater glider [13,18,19,20]. ROUGHIE is affordable at 10% of the cost of commercial underwater gliders. The low weight and petite frame (12 kg and 1.2 m) ensure the ROUGHIE can be deployed and retrieved by one person, either by boat (with no additional equipment required) or from shore. The low-cost of ROUGHIE enables the deployment of disposable gliders on experimental or dangerous missions. ROUGHIE’s modularity allows for a wide range of sensors to be installed and implemented for many different end uses.

Localization of underwater vehicles while underway typically relies on dead- reckoning [21], with some groups exploring novel augmentations [22]. Regardless of the method, no solution exists to enable millimeter level accuracy when localizing in open water. One alternative in enclosed water is the use of underwater motion capture such as the Qualysis™ underwater motion capture system. The underwater motion capture system operates on the same principle as aerial motion capture systems [23] and is able to achieve ground truth accuracy on the order of a few millimeters at 100 Hz. This accuracy is useful from a research perspective as it effectively eliminates any uncertainty in vehicle position while interpreting the collected magnetic data.

Industry standard magnetic survey systems of the early 21st century [6,24] have dimensions on the order of a meter, and even the smallest systems weigh nearly 2 kg per sensor. Such instruments are limited in range by the vehicle which provides locomotion. Given that the energy required to move such sensors through the water is significant and they draw tens of watts of power during operation, many smaller AUVs and ROVs are unable to support the necessary equipment for extended missions.

As AUVs have advanced in recent years, so have compact, low-power optical magnetometers [25]. These systems have enabled the utilization of small AUV/ROV systems for magnetic surveys [4]. Optical magnetometers offer high-precision, calibration-free measurements of the Earth’s magnetic field and distortions of that field due to the presence of magnetic objects. The operational concept of optical magnetometers is well established, but recent developments in both physics approach and technology [25] have enabled new applications using sensors that are smaller, lower mass, and lower power.

A standard approach to an optically-pumped scalar atomic magnetometer uses a control loop to lock the resonant frequency of spin-polarized atoms in a magnetic field. This frequency is determined by a fundamental physical constant, providing a direct measurement of the local magnetic field. Different types of optical magnetometers can provide scalar or vector field measurements. Multiple sensors can be configured into a gradiometer capable of measuring the total or partial gradient in the local magnetic field. Gradiometric measurements provide common mode noise subtraction, but have an operating range dependent on the separation and arrangement of the sensors.

The chief design challenge for successful magnetometry on any moving platform (AUV or ROV) is maintaining magnetic signal integrity in close proximity to the platform itself. The sensitivity of magnetic detection, i.e., the range at which a given magnetization can be distinguished from background noise, is limited in part by the magnetic noise imposed by the platform and its interaction with the environment. Platform-related noise can manifest in a number of ways, including: AC signals due to motors and other electronics, eddy currents generated by conductive components moving through Earth’s field, and any overall permanent magnetization of the platform. Any contribution from these noise sources can increase the noise floor over various frequency bands, and limit the sensitivity for detection.

Platform sources of magnetic noise can be reduced by keeping the magnetometer far from the noise source (e.g., on a tow cable [26]), but this increases the size of equipment required, reduces maneuverability, and increases the power consumption due to locomotion. Software compensation can be effective at reducing motion-related platform noise due to platform static magnetization, induced magnetization, and eddy currents [4,17].

Environmental noise also limits detection sensitivity. Estimates of ocean wave noise range from about 25 pT [27] to 1 nT [28]. The motion of conductive seawater can also generate apparent fields and gradients on the order of 100 pT and 100 pT/m [29]. One review of undersea magnetometry asserted that the smallest practical level of reliable anomaly detection was 5 nT, despite the use of instruments with a far lower noise floor [30]. Where possible, an AUV can operate far below the waves and thereby avoid some of this noise source.

## 3. Vehicle Setup

To experimentally validate the effectiveness of utilizing a compact quantum magnetometer on an agile underwater glider, the authors modified a custom underwater glider, ROUGHIE. A detailed discussion of the platform [13], controller design [20], and operation [18,19] are available in prior publications.

The vehicle was modified to operate with a large sensor boom attached to it, which housed a sensor suite comprising of an optical scalar magnetometer and a magneto-restive vector magnetometer. A test procedure was then developed to validate the ability of the system to detect a localized magnetic anomaly within a testing environment with a high noise floor.

### 3.1. Underwater Glider

**Figure 2:**
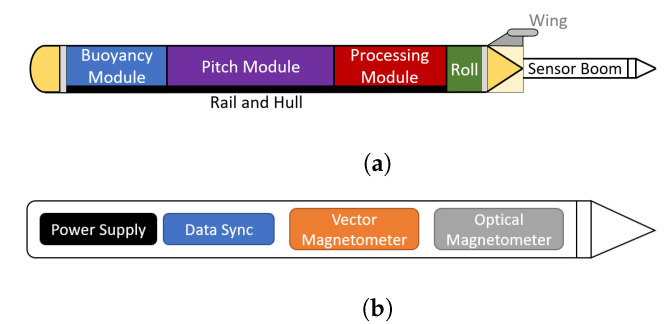
Block diagram of ROUGHIE showing control modules utilized for vehicle control (**a**). Aft of the vehicles stern is the sensor boom, comprising all of the magnetic sensing equipment (**b**).

ROUGHIE is a small, highly-maneuverable, and low-cost underwater glider [13] that is controlled by a frontseat-backseat architecture similar to other underwater vehicles [31]. As with other underwater gliders, ROUGHIE utilizes a ballast volume to create changes in buoyancy and enact changes in depth/altitude. A slide system mounted internally allows mass to be moved forward and aft to control the vehicle’s pitch during ascent and descent maneuvers. To turn, ROUGHIE uniquely mounts nearly all internal components on a single rail which can be rotated within the hull to initiate a banked turn. This results in a tight turning radius of approximately 3 m [20], but makes internal mounting of sensing equipment difficult. To solve this issue and further distance the sensor from any noisy actuators, magnetic sensing equipment is mounted externally and aft of the vehicle in a sensor boom (Figure 2a).

To reliably measure magnetic anomalies in the water, the vehicle must not exhibit a significant magnetic signature. The ROUGHIE was modified to reduce its magnetic signature. All internal structural components are replaced with non-ferrous materials, principally Aluminum 6061. Servos used to roll the center of gravity of the vehicle to enact a maneuver typically must be powered to maintain the desired roll angle. Using a servo motor that exerts rotational movement through a worm gearbox permits disabling the servo when not in use. Similar to [32], a non-magnetic carbon fiber composite hull also provides minimal eddy current magnetic noise when compared with standard metallic hulls. Example data from these efforts is shown below in Section 5.

The sensing boom is an isolated pressure vessel that houses all components necessary for magnetic sensing and is placed aft of the stern of the vehicle. The boom is 65 cm long with an internal diameter of 5 cm. As shown in Figure 2b, the boom houses a power supply, a vector magnetometer, optical magnetometer, and a small micro-controller to log and time stamp the data from the two magnetometers. Pairing the vector and optical magnetometers in this way provides total system magnetic heading errors. Despite the added buoyancy caused by the sensor boom, ROUGHIE is robust enough to maintain high performance by mounting a counterweight on the boom to negate the added buoyancy due to the boom.

In addition to its low overall magnetic signature, ROUGHIE has unique capabilities that make it suited to magnetometry. Unlike ocean gliders, ROUGHIE has the ability to change its flight characteristics and enter a stable glide quickly, thus allowing the vehicle to operate in shallow confined testing environments. The vehicle’s ability to quickly change flight characteristics and enter into a stable glide rapidly is due to several key design and controller features [13,20].

A significant portion of ROUGHIE’s mass is movable along the vehicle’s primary axis and with relatively rapid actuation. This allows the vehicle to change its pitch angle quickly. However, such a change in flight characteristics leads to non-linear responses from the vehicle which makes feedback control difficult. Utilizing feed-forward control, ROUGHIE positions internal mass and buoyancy tank levels to a preset point to achieve a desired glide angle during non-linear transition periods. This allows ROUGHIE to enact stable and controlled glides in shallow water.

**Figure 3:**
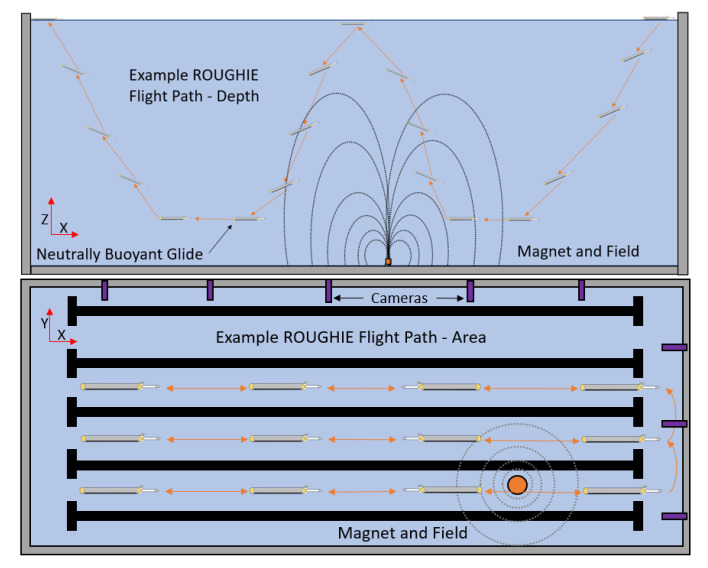
Illustration showcasing a single idealized example ROUGHIE data collection flight path across the testing volume (**Top**) and an Illustration showing an idealized lawnmower coverage path by the vehicle through the entire testing area (**Bottom**).

Similarly, at the end of each glide cycle, the vehicle remains in level flight for a set time before beginning another ascent/descent. Neutrally buoyant dwell maneuvers improve sensing resolution at a target depth as the vehicle can hover at an optimal depth for extended periods of time, gliding and sensing, before an ascent maneuver is required (illustrated in Figure 3). This is crucial for magnetic sensing applications that require constant distance between the sensor and a potential magnetic anomaly, while maintaining a constant speed in level flight. Additionally, the neutrally buoyant maneuver helps to minimize pitch overshoot, resulting in faster convergence to steady gliding motion.

The heightened maneuverability of the ROUGHIE in confined environments enables the traversal of the entire water column depth (5 m) of the confined testing environment twice in a distance of only twenty-five meters. This increased depth coverage allows ROUGHIE to gather enough data to observe the magnetic field gradient present in both XY and XZ planes (Relative to Figure 3 in shallow environments).

The use of a magnetometer aboard ROUGHIE enables an unprecedented low-power, high endurance, and high spatial resolution underwater magnetic exploration and mapping system. The vehicle is well suited to perform many sensing missions, even in the confined testing environments of shallow/coastal water, where vessels and towed arrays have limited maneuverability. ROUGHIE’s ability to enact controlled, repeatable dives in shallow water while being able to dwell at depth enables the vehicle to target localized magnetic anomalies in areas smaller than a square meter. ROUGHIE’s unique ability to complete turns within a 3 m radius also means that the vehicle can revisit small localized areas rapidly.

### 3.2. Sensor Suite

The magnetic boom in this work is composed of a Twinleaf compact optical scalar magnetometer, magneto-resistive vector magnetometer, and a synchronization unit, all powered by a rechargeable lithium ion battery. The sensors and wire harness are rigidly mounted in 3D printed holders which can be positioned on fiberglass rods. The entire assembly, with a power supply, was sealed in a pressure vessel for attachment to the ROUGHIE.

The optical scalar atomic magnetometer used in this work has a 20 pT/$\sqrt{\mathrm{Hz}}$ noise floor. The sensor employs microfabricated rubidium vapor cells and a VCSEL laser to interrogate precessing atomic spins. The sensor provides a calibration-free readout of the total magnetic field by measuring the rubidium atoms’ magnetic resonance frequency, which is proportional to the magnetic field by a fundamental constant. The sensor is run using the M${}_{z}$ operating mode.

The companion vector magnetometer used in this work is a 3-axis magnetoresistive vector magnetometer, combined with an Inertial Measurement Unit (IMU) and pressure/temperature logging. It provides valuable insight into the measurement conditions and records data to compensate for systematic errors in the total field measurement.

Both sensors were configured to collect data at 200 Hz for this experiment. The data streams are given a synchronous clock by the Twinleaf SYNC2 synchronization module, so that the data is collected simultaneously from both sensors. The unit also distributes power, and logs the results to an SD card for later extraction. The entire system consumes less than 1.5 watts of power, and can run for several hours on a single charge of the integrated battery.

## 4. Test Procedure

The objective of system testing is to use the ROUGHIE to locate a magnetic target sitting on the bottom of a pool. All system testing took place at Purdue University’s Morgan J. Burke Aquatic Center’s Diving Well. The diving well is 22.9 m by 17.37 m with depths ranging from 4.25 to 5.18 m encompassing an area of 398 square meters and volume of 1.95 million liters.

ROUGHIE position data within the test volume was collected using a Qualysis™ underwater motion capture system. Qualysis cameras triangulate infrared (IR) reflective marker positions on the underwater glider through the use of infrared emitters and sensors. The system outputs rigid body 6-axis (X, Y, Z, Pitch, Yaw, Roll) positions at 100 Hz with high accuracy of approximately 3–5 mm. One of the markers was placed at the end of the magnetic boom, within ∼5 cm of the scalar magnetometer sensing volume. The position of that marker is used for the data presented here.

The target consists of a neodymium magnet (K&J Magnetics p/n DZ0X8-N52) secured within a capped section of standard 10.18 cm (4 inch) PVC pipe. The target’s magnetic field is oriented such that when placed upright on the bottom of the pool, the target’s field adds to the Earth’s magnetic field in the region above the target. The pool has a large background magnetic signature so mapping is performed first without the magnetic target and second with the magnetic target. This resembles many littoral environments with magnetic clutter on the seafloor and indicates the ability of an autonomous vehicle on routine patrol to detect magnetic changes.

The first stage is the magnetic baseline characterization of the test volume. Without the target present in the testing environment, the glider performs parallel tracks across the pool (along the X-axis in Figure 3) covering a length of the pool in both directions before moving onto the next track all while diving through the test volume. Each path started with the ROUGHIE on the surface of the testing environment. A manual turnaround process is used due to the space-constrained pool environment. The data gathering path is illustrated with the target present in Figure 3. The start of each run is marked on the side of the testing volume (y axis in Figure 3) so that it is repeatable.

Upon the completion of the first stage, magnetic targets are seeded on the bottom of the pool. At least one magnetic target is placed so that it is coincident with one of the parallel tracks. The system proceeded to collect the same data along the same tracks for a second time.

Position data from the motion capture system is aligned with the magnetic field data through manual timestamp synchronization in post-processing. The magnetometer system presently runs on a clock independent of the ROUGHIE’s telemetry and the mocap system. We were able to align the timestamps by coordinating three data streams. First, the ROUGHIE telemetry provides a depth measurement, which was used to match the clocks between the ROUGHIE and the Qualysis position data. Then the ROUGHIE data provided a means to sync the vector magnetometer/IMU clock in the magnetometer boom. Finally, the common time axis was confirmed by matching observations from the vector magnetometer to the position data. Magnetometer data was downsampled and interpolated to the position data for plotting. The combination of both systems allows magnetic field data to be paired with a Cartesian point within the testing environment at which the magnetic field strength and direction was observed. The resulting 3-D data can be used to identify and visualize changes in the magnetic environment caused by a target.

## 5. Results

Experimental evaluation of the feasibility of using the ROUGHIE for magnetometry was completed in two phases. In phase one, the ROUGHIE was deployed at the Purdue University Morgan J. Burke Aquatic Center to evaluate if the ROUGHIE was physically capable of hauling the magnetometer and if detection was possible. In phase two, the ROUGHIE design was modified slightly to reduce the vehicle magnetic signature. Reducing the vehicle signature enables detection of smaller targets at further distances.

### 5.1. Data Collection

**Figure 4:**
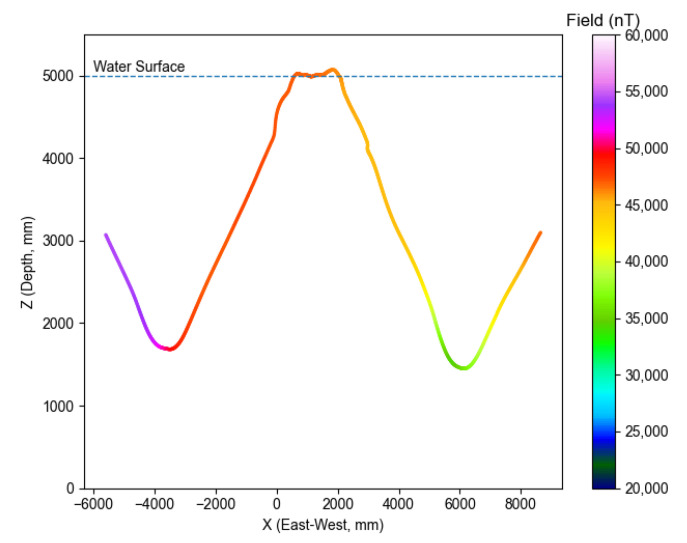
A typical track, viewed from the south wall of the pool.

**Figure 5:**
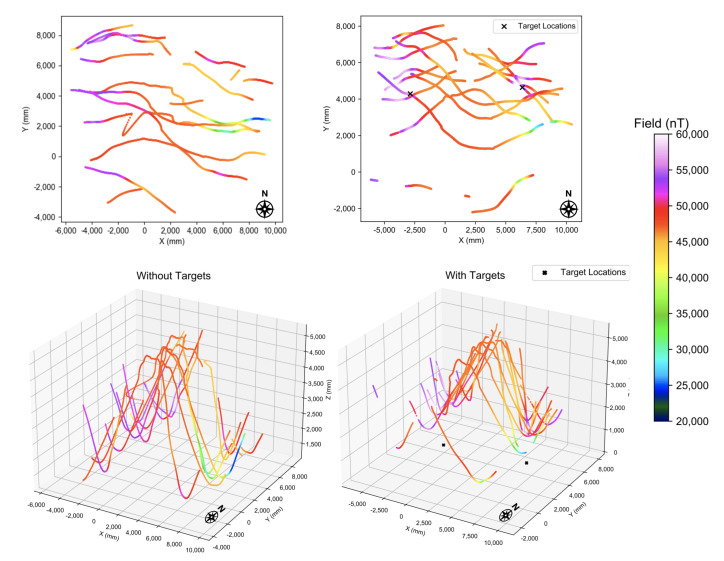
Glider trajectory for background and experimental tracks, colored according to scalar total field. Magnetic targets were installed at the dark points on the bottom of the pool. The experimental analysis focuses on the target on the right side.

The ROUGHIE was initially deployed into the Purdue pool to evaluate the feasibility of performing magnetometry with the ROUGHIE and Compact Quantum Magnetometer. This testing had two focuses, to validate that the vehicle is physically capable of hauling the sensor boom and to validate that the sensor boom will be capable of sensing in the presence of disturbances from the vehicle and environment. The addition of the sensor boom doubled the length of the vehicle, and significantly altered the center of gravity of the ROUGHIE. After re-trimming the vehicle, ROUGHIE was still able to effectively locomote through the testing environment as shown in Figure 4 and Figure 5. The ROUGHIE glider equipped with the sensor boom completed eight nominally east to west (X-axis) sawtooth characterization tracks across the testing environment with and without magnetic target seeding.

Figure 4 shows a typical track in the pool without any targets seeded. Trajectory data is plotted for the XZ plane, giving a view from the south wall of the pool. The trace is colored according to the total scalar field. As the magnetometer approached the bottom and sides of the pool it encountered significant magnetic gradients. These fields are likely due to magnetic components used in the pool’s construction. The field within the pool’s volume varied considerably, from roughly 25 μT to 60 μT.

Extending this example, we can represent the data for both phases as shown in Figure 5. Full 3D trajectories are shown, colored by the scalar field strength. Empty points within the data set represent locations where the motion capture system IR reflectors were out of the camera’s view and thus prevented positioning of the vehicle and magnetic boom. Presented also are the locations where the magnetic targets were placed.

In 3D space the tracks reveal the overall magnetic landscape of the pool. The East side of the pool, and especially near the bottom, is at a significantly higher field than the rest. There also appears to be a strong low-field anomaly near the center of the west end. These fields were present in the pool as a baseline, prior to any test targets being seeded.

The magnetometer data presented here is uncorrected for the heading error. Although the magnetometer boom is equipped with a vector magnetometer for making heading corrections, we found that the gradients in the pool precluded any accurate heading analysis. Nevertheless, the pool’s field variations were by far the dominant effect on the sensor, and they varied beyond the limits that we would expect given platform magnetization and sensor heading error. In future open water tests, heading error correction will be applied.

Throughout the data presented, the uncertainty in position is smaller than the points used to represent the data. The uncertainty in field measurements is dominated by gradients in the environment and residual magnetism in the platform, but the target was chosen so that the signal is clearly visible above background noise.

### 5.2. Target Detection

The same nominal set of tracks were completed before and after targets were added, Figure 5. The glider was released in the same starting location for each track, and the internal program governing the maneuvers was the same. However, The trajectories do not perfectly overlap between the two runs, due to the effects of underwater disturbances nudging the glider off of its straight course and the lack of vehicle position feedback in the pool environment. Due to the constrained space of the testing environment, a manual turnaround procedure was completed at the end of each track.

**Figure 6:**
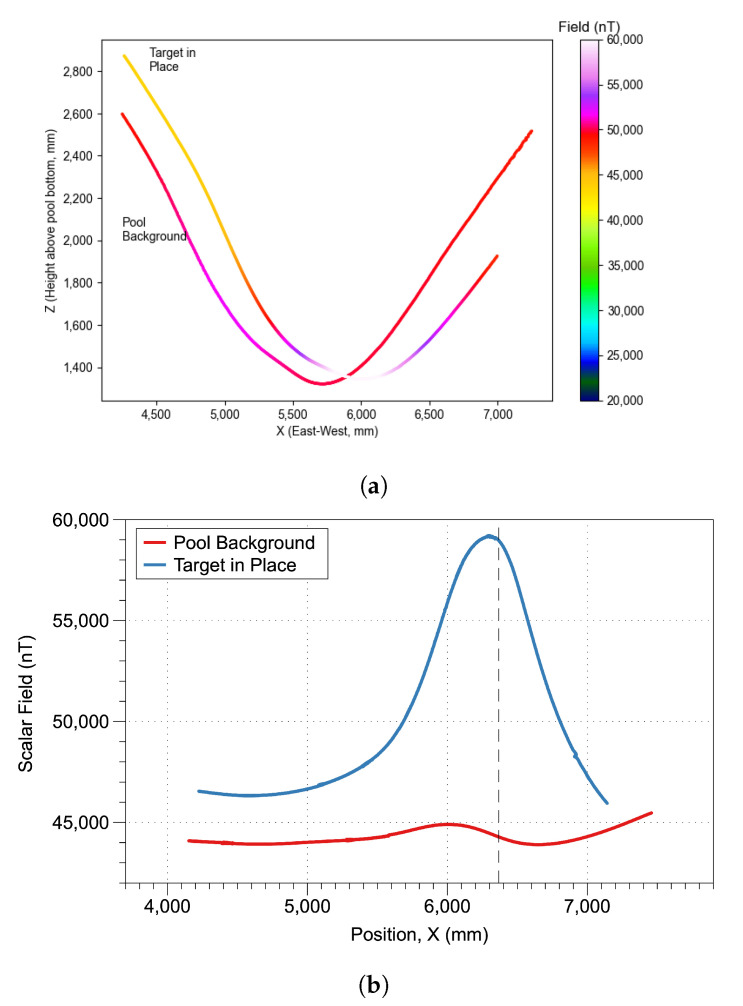
Comparison of glider flight tracks before and after target seeding in the testing environment. Localized changes in magnetic scalar field along East-West glider tracks (X-axis) shows the viability of target detection with the underwater glider platform (**a**) Two glider tracks within 0.5 meters y from the background and experimental tracks. Traces are colored according to scalar total field. (**b**) Scalar field magnitude before and after target seeding.

To evaluate feasibility of detection, a direct visual comparison of the scalar data from two similar tracks (before and after seeding) was completed, Figure 6. In Figure 6a the ROUGHIE traveled along approximately the same trajectory on both tracks which went directly over the seeded target. When the raw data is correlated to the X position (Figure 6b), the magnetic target is more obvious with a dramatic dipole around the target location. Although the two tracks do not perfectly overlap, the size and distribution of the anomaly is significant enough that the anomaly would clearly have been visible in a single data gathering trace.

This detection is a proof of principle for the viability of this platform. The dive pool used here presented a confined space too small for a comprehensive grid search pattern. This precluded localization of the test target along the y-axis (North-South). Additionally, the background magnetism in the pool varies dramatically as shown by the initial survey necessitating the use of a large magnetic target.

Future outdoor testing in larger bodies of water will allow for the completion of significantly longer tracks covering a wider search area, allowing for the location and characterization of spatially larger anomalies. Outdoor testing will also enable intermittent vehicle localization feedback from Ultra Short Baseline (USBL) or surface measurements, enabling finer vehicle control. Furthermore, in a real environment, at-sea or littoral, the background gradient would be significantly lower and magnetic anomalies of much lower magnitude would be visible in the data.

### 5.3. Mitigating Glider Magnetization

**Figure 7:**
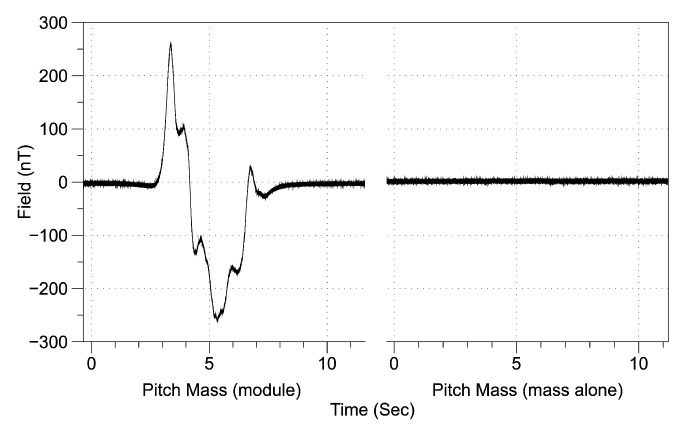
Magnetization test data for ROUGHIE components.

To increase the usability of the ROUGHIE on magnetometry missions, the vehicle has been demagnetized in two stages. The first stage addressed ferromagnetic parts with strong magnetization and was completed prior to experimental testing presented earlier. A Twinleaf microSAM magnetometer was fastened in place, the ROUGHIE was systematically dismantled, and every component was passed at approximately 10 cm from the sensor on a linear path. The ROUGHIE’s components were tested as assemblies first, and then each assembly was further dismantled to discern which parts, in particular, might be magnetic. For example, Figure 7 shows the magnetization of the entire pitch mass module, versus the pitch mass itself, revealing a magnetic component in the module. The majority of the existing ROUGHIE components were already non-magnetic or had a minimal signature, however, some components such as the rail ends were replaced with aluminum versions.

**Figure 8:**
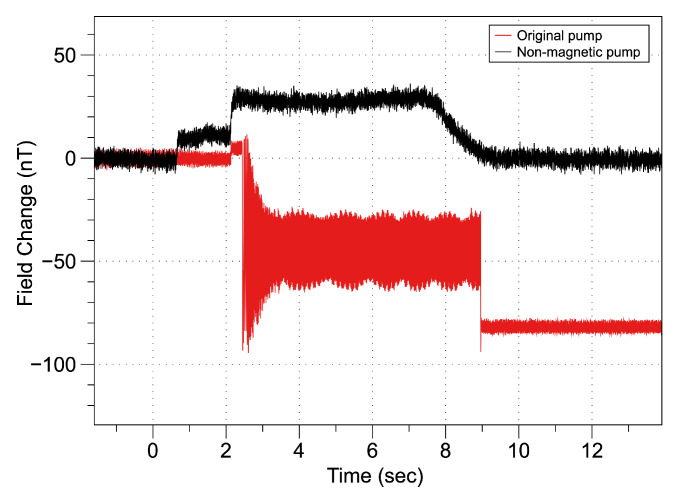
Magnetic fields created during the operation of ROUGHIE’s ballast pump. Note that the original pump had a magnetization that would land in an arbitrary position, resulting in an unpredictable offset to the field. The new non-magnetic pump however, returns to the same field strength upon being depowered.

In the second stage of demagnetization, the microSAM was positioned aft of the ROUGHIE and the glider’s various motors and servos were energized. This provided information about dynamic magnetic fields that may be present during operation. During this phase of testing, it was found that the ballast pump was an important source of unpredictable magnetic field noise. Critically, in addition to the fields produced by energized wires, the ballast pump had a DC magnetization that moved during operation and landed in a random position likely caused by the magnetic coupling between the brushless pump motor and pump head. An example of this behavior is shown in Figure 8, in which the pump lands in a position that offset the local field ∼80 nT from its original value. This was not remedied for the presented results, however, due to the high noise floor in the testing environment, it did not present an issue in analyzing the results. Overall, vehicle disturbance is on the order of 10 s to 100 s of nT in the presented results while the seeded anomaly is on the order of 10,000 nT. Once the pump noise issue has been resolved, identification of targets in the lower noise open water environment should be possible.

## 6. Conclusions and Future Work

In this paper, we have presented an agile underwater glider equipped with a magnetometer boom. ROUGHIE’s modular nature allowed for extensive demagnetization of the entire submersible. The low magnetic signature and high maneuverability of ROUGHIE makes it an excellent platform on which to conduct magnetometry experiments and surveys. This will enable detailed magnetometer surveys to be completed at greatly reduced cost and difficulty over more traditional ship-based, ROV-based, and large AUV-based surveys. Integration testing in an indoor pool shows strong promise for future endeavors.

To allow detection of low magnitude magnetic anomalies in open water environments the randomized magnetic noise from the pump must be eliminated. To achieve this a custom nonmagnetic replacement pump utilizing no permanent magnetic components was manufactured, and tested. The improvement over the brushless pump motor and the head is shown in Figure 8. The random offset effect has been corrected, and while there is still some field created during operation, both the DC and AC components are greatly reduced compared to the original pump. Without a randomized DC signature, the affect of the pump on the vehicle’s overall magnetic noise can be filtered from sensing data, enabling greater sensing precision upon the pumps integration with the vehicle.

**Figure 9:**
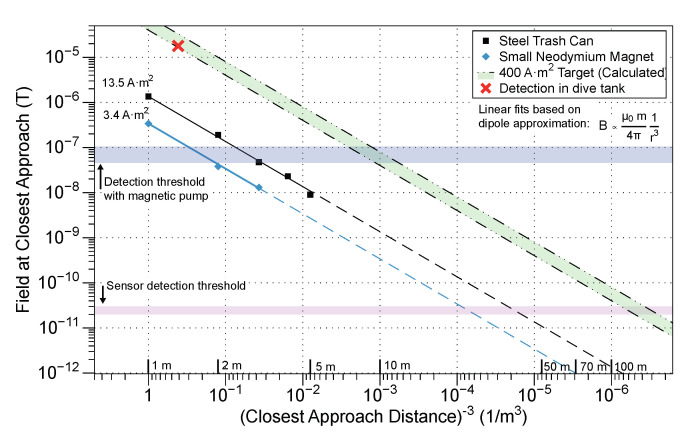
Reference data for detection capabilities. The maximum range of the sensor varies strongly depending on both the magnetization of the target and its distance from the sensor, since the field decreases inversely proportional to the cube of distance.

Going forward, we intend to leverage the ROUGHIE vehicle’s modularity to develop a new system that features effectively no detectable magnetic noise to enable the integration of the magnetometry equipment into the main vehicle hull. This will create a more maneuverable and robust platform for use in underwater magnetic sensing applications. In addition, decreasing the platform magnetic noise will allow for leveraging of the sensor’s full sensitivity. Figure 9 provides context for the broader goal of target detection. Modeling targets as magnetic dipoles allows for an estimate of detection capability. A “detection” here is defined as a change in a field that would be visible above noise. Data for two real objects, a trash can and a neodymium magnet the size of a AA battery, were collected with a Twinleaf microSAM. Calculated data for the pool target, as well as the point representing the above detection, are also shown.

Reducing the amount of onboard magnetic interference will result in an improvement of detection capability by three orders of magnitude.

Future work includes open water testing and on-vehicle real-time data analysis. Open water testing will provide an environment with a lower magnetic noise floor, and possibly allow for localization of a target without the need for baseline calibration. Doing on-vehicle real-time data analysis will allow real-time path planning and tracking to identified magnetic anomalies. Integrating multiple sensors into a single-vehicle will allow for gradiometric measurements, which can increase noise rejection and allow for the detection of more diverse targets.

To pursue real-time tracking, underwater navigation, and submerged communication, an Ultra-Short Baseline (USBL) acoustic modem system has been implemented on the ROUGHIE. The system provides position feedback and real-time wireless underwater communication with the vehicle. Preliminary testing of the system has provided submerged localization in open water and communication. These open-water functionalities will allow ROUGHIE to locate, track, and characterize underwater magnetic anomalies with real-time user feedback in future endeavors. Fleets of ROUGHIEs equipped with magnetic sensing equipment will increase the rate of data collection and effectiveness of anomaly detection on large area missions.

Long-term, this work can form the basis for the creation of a persistent marine sensing network capable of autonomous search, exploration, and mapping of magnetic anomalies. The described marine sensing technology, when combined with real-time communications will enable timely automated detection and classification of underwater objects. Further, the glider/magnetometer duo can be combined with other emerging technologies such as deep learning to enable enhanced detection and classification of marine objects through multidimensional signal processing.

## Data Availability

The data presented in this study are available on request from the corresponding author and Twinleaf LLC. The data are not publicly available due to proprietary restrictions on distribution of magnetometer data.
